# A comprehensive study on the impact of human hair fiber and millet husk ash on concrete properties: response surface modeling and optimization

**DOI:** 10.1038/s41598-024-63050-7

**Published:** 2024-06-12

**Authors:** Naraindas Bheel, Muhammad Alamgeer Shams, Samiullah Sohu, Abdul Salam Buller, Taoufik Najeh, Fouad Ismail Ismail, Omrane Benjeddou

**Affiliations:** 1 Department of Civil and Environmental Engineering, Universiti Teknologi PETRONAS, Bandar Seri Iskandar Tronoh, 32610 Perak, Malaysia; 2 Department of Civil Engineering, The University of Larkano, Larkana, Sindh Pakistan; 3 Department of Civil Engineering (TIEST), NED University of Engineering and Technology, Karachi, Sindh Pakistan; 4 Operation and Maintenance, Operation, Maintenance and Acoustics, Department of Civil, Environmental and Natural Resources Engineering, Luleå University of Technology, Luleå, Sweden; 5 Department of Civil and Environmental Engineering, University of Nebraska-Lincoln, 1110 67Th Street, Omaha, NE 68182-0178 USA; 6 Department of Civil Engineering, College of Engineering, Prince Sattam Bin Abdulaziz University, 11942 Alkharj, Saudi Arabia

**Keywords:** Concrete, Millet husk ash, Human hair fiber, Hardened properties, RSM modeling and optimization, Engineering, Materials science

## Abstract

Revolutionizing construction, the concrete blend seamlessly integrates human hair (HH) fibers and millet husk ash (MHA) as a sustainable alternative. By repurposing human hair for enhanced tensile strength and utilizing millet husk ash to replace sand, these materials not only reduce waste but also create a durable, eco-friendly solution. This groundbreaking methodology not only adheres to established structural criteria but also advances the concepts of the circular economy, representing a significant advancement towards environmentally sustainable and resilient building practices. The main purpose of the research is to investigate the fresh and mechanical characteristics of concrete blended with 10–40% MHA as a sand substitute and 0.5–2% HH fibers by applying response surface methodology modeling and optimization. A comprehensive study involved preparing 225 concrete specimens using a mix ratio of 1:1.5:3 with a water-to-cement ratio of 0.52, followed by a 28 day curing period. It was found that a blend of 30% MHA and 1% HH fibers gave the best compressive and splitting tensile strengths at 28 days, which were 33.88 MPa and 3.47 MPa, respectively. Additionally, the incorporation of increased proportions of MHA and HH fibers led to reductions in both the dry density and workability of the concrete. In addition, utilizing analysis of variance (ANOVA), response prediction models were created and verified with a significance level of 95%. The models' R^2^ values ranged from 72 to 99%. The study validated multi-objective optimization, showing 1% HH fiber and 30% MHA in concrete enhances strength, reduces waste, and promotes environmental sustainability, making it recommended for construction.

## Introduction

The increasing demand for building constituents owing to the increase in construction activities has produced significant pressure on the supply of natural resources needed for producing concrete. The rapid depletion of natural resources has forced civil engineers and researchers to find alternative materials to utilize in concrete production^1–4^. Researchers can use waste by-products as substitute materials, but they face the challenge of transforming agricultural and industrial wastes into useful structural elements for the construction sector^5^. The purpose of management is to reduce the hazardous impact of waste by-products on the health and well-being of the environment^6^. The significance of concrete manufacturing lies in its high energy consumption, which consumes considerable natural resources, as well as its capacity to use waste materials^7^. The production capacity of a concrete factory is around 8–10 tonnes of freshly mixed concrete each day^8^. The selection of polymers is crucial, and their significance is clearly evident in limiting the strength of concrete and degrading its stability and performance due to their properties^9^. The worldwide consumption of sand in concrete structures is extremely high, and it is increasing every year. Developing countries face enormous pressure to keep supplying natural sand for the growing infrastructure construction^10^. To reduce the pressure and demands of river sand, scientists have acknowledged alternative sands such as industrial waste, including tile powder^11^, silica fume, marble powder^12^, and ground granulated blast furnace slag, while agricultural discarded resources like wheat straw ash, rice husk ash^13^, fly ash^14–16^, groundnut shell ash, maize cob ash^17^, coal bottom ash, and millet husk ash (MHA).

Owing to environmental problems unavoidably associated with social and economic characteristics, environmental oversight in developing countries faces many complex problems that must be considered when developing an environmental plan or directive^18–21^. Globally, there is a significant generation of waste materials, especially in densely populated zones^22^. The reprocessing of solid agro-industrial waste as a material for sand production in construction projects saves landfills and reduces the necessity for the extraction of natural raw resources^23^. As a result, this study aims to replace sand with millet husk ash in cement concrete production. The millet crop belongs to the family of small-seeded grass cereal crops grown across the world. It forms numerous small particles that arise in a diversity of unfavourable circumstances, generally in dry, semi-arid, and semi-humid agro-ecosystems. However, millet grows in nine forms, with a total production of 28.38 million tonnes worldwide, of which 4.53 tonnes are generated in Nigeria and 11.36 tonnes are created in Africa^24^. On 5.8 million hectares, the millet yield is about 6.7 million tonnes^25^. Due to insufficient rainfall and unfavourable weather conditions, the northern region produces more than 80% of the millet. Nigeria ranks as the world's second-largest manufacturer of millet^26^. The millet seeds and grains taken out of the millet husk^27^ have no additional use and are regarded as agricultural waste, so they are dumped into a sewage pond as a landfill or as organic fertiliser spread on farms that decompose and turn into the soil over the years with limited fertiliser efficiency. Millet husk ash (MHA) is formed when millet husks are burned under controlled temperatures and conditions. The ash can be used as a substitute for sand in the production of concrete mixtures to decrease the usage of natural sand in construction and boost the use of agricultural residues for commercial purposes. A comprehensive economic study is necessary to determine the viability of using MHA as a substitute for sand in concrete. Although MHA is derived from waste sources, it still involves expenses for transportation and processing, such as drying, crushing, and sieving. The cost of the method is $0.0048 per kilogram, in contrast to $0.009 per kilogram for sand. Hence, MHA proves to be a more economical resource for concrete manufacturing in comparison to sand since it is derived from waste sources without incurring direct procurement expenses. Moreover, it has been found that the MHA possesses pozzolanic properties, thus triggering a pozzolanic reaction during which free accessible Ca(OH)_2_ can be used to produce secondary calcium-silicate-hydrate (C–S–H)^28^, which results in improving the hardening characteristics of concrete.

Though substituting natural sand content with MHA may improve the overall hardened characteristics of concrete; however, it is still relatively weak under tension^29^; therefore, to overcome this deficiency by utilising several kinds of fibers. Over the years, various fibers such as human hair fibers^30^, coir fibers^31^, Forta-Ferro fiber^32^, polypropylene fibers^33,34^, nylon fibers^35^, jute fibers^36^, carbon fiber^37^ and steel fibers^38–44^, etc., develop fibers reinforced concrete; however, the current experimental study focuses only on the utilization of human hair fibers as a reinforcement material. The utilization of fibers as reinforcement material in concrete has gained significant importance because steel bar reinforcement in concrete poses a substantial risk of corrosion, which damages concrete. However, Fiber-reinforced concrete (FRC) is a composite material that combines the strength and durability of conventional concrete with the improved characteristics provided by different fiber kinds^45–47^. The fibers, often composed of elements like steel, glass, synthetic polymers, or natural fibers, are evenly dispersed inside the concrete mixture to enhance its performance and toughness. Accumulation of fibers to composites helps reduce cracking, boost tensile strength, and boost the overall durability of the material^48,49^. As a consequence, this leads to a substance that can endure higher amounts of pressure, demonstrate stronger resilience against impact and fatigue, and provide superior durability under challenging environmental circumstances^50,51^. FRC is utilized in various construction projects, such as bridges, pavements, and high-rise buildings, because of its exceptional mechanical qualities that enhance the durability and structural stability of the structure.

The Sustainable Development Goals can meet present desires and necessities without risking future generations. Nowadays, massive population growth has led to a large amount of human hair waste. Waste generated by human hair is disposed of in open landfills. It is measured municipal solid waste (MSW) in most areas of the world which can be obtained as solid household waste^52^. In most developing countries, human hair is either burnt with solid household waste or thrown into the sewer and dumped down the drain through the toilet, which has an adverse impact on the environment. Human hair clogs pipes and raises nitrogen levels in sewerage when it is thrown down the drain, and finally, the sewage is poured out into the street through a hole in the sewer. The human hair is dumped under the atmosphere, dust will build upon it, which can cause unrest in neighbouring houses. Hair waste is discarded openly in sparsely populated areas, taking years to decompose. Burning human hair can cause an unpleasant odor and release harmful gases such as sulphur dioxide, ammonia, pyrite, sulphides, phenolic, nitrogen, pyrrolidine, and benzoic^53,54^ causing ecological degradation. Hair on the head grows at a pace of roughly 12.7 mm per month, and its weight ranges from 80 to 100 g per year^52^. Considering that the overall global population is 7.7 billion^55^, approximately 6.9 × 10^5^ tons of hair are produced annually. It has been conducted a rough inspection among the workers of beauty Parlors and saloons about the disposal of hair waste and most beauty salon and barbershop workers agreed that there is no efficient way to dispose of non-biodegradable hair, so they have just been throwing it away. Therefore, this study is an investigative one to assess the potential of human hair as a fiber in concrete, and the finding if positive will create awareness of human hair waste usage leading to further research in the area with the possibility of establishing a structured collection, processing, and usage that are presently lacking.

Furthermore, the same amount of hair has been discarded as MSW in the past few years. As a result, its use in the construction industry is getting increasing attention. Using hair waste in concrete restricts the dumping rate and provides supplementary benefits such as energy-saving and environmental safety. Besides, human hair (HH) fibers, when used to reinforce the material in cement concrete, decrease the brittle behaviour of concrete^56^. The utilization of discrete fibers improves brittle complexes in an olden perception. However, during the nineteenth century, fibers were first used in concrete to improve its flexibility and resistance to fractures^57,58^. Fibers in concrete can improve tensile and flexural strength^57,59–64^, slow crack propagation and increase durability^54^. Fibers provide the capacity to effectively control fractures that result from both plastic and drying shrinkage^57,65–67^. Most studies^68–70^ have been performed on human hair (HH) as fiber reinforcement in concrete. However, to the best of the authors' knowledge, millet husk ash (MHA) as a sand substitute component had not been used to explore the workability and hardened characteristics of concrete, and no investigation was achieved on concrete combined with different concentrations of HH fibers along with MHA as a sand replacement ingredient. Therefore, the goal of this study was conducted on concrete reinforced with various HH fibers and MHA ratios separately and in combination to determine the workability, dry density, compressive strength, and tensile strength of concrete by using the response surface methodology (RSM) tool to model and optimize the input factors (MHA and HHF).

## Experimental procedures

### Materials

**Figure 1:**
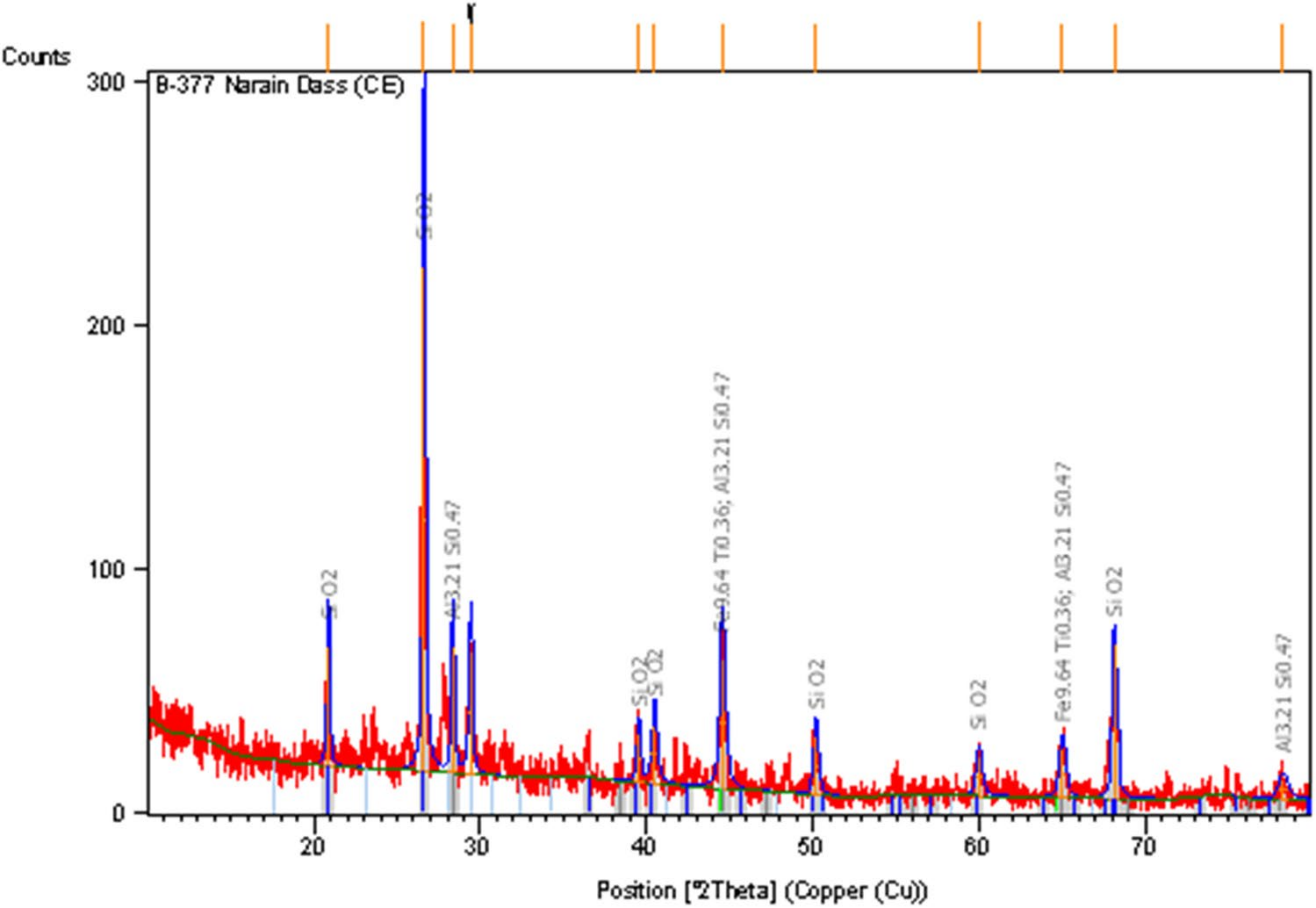
XRD of MHA^71,76^.

**Figure 2:**
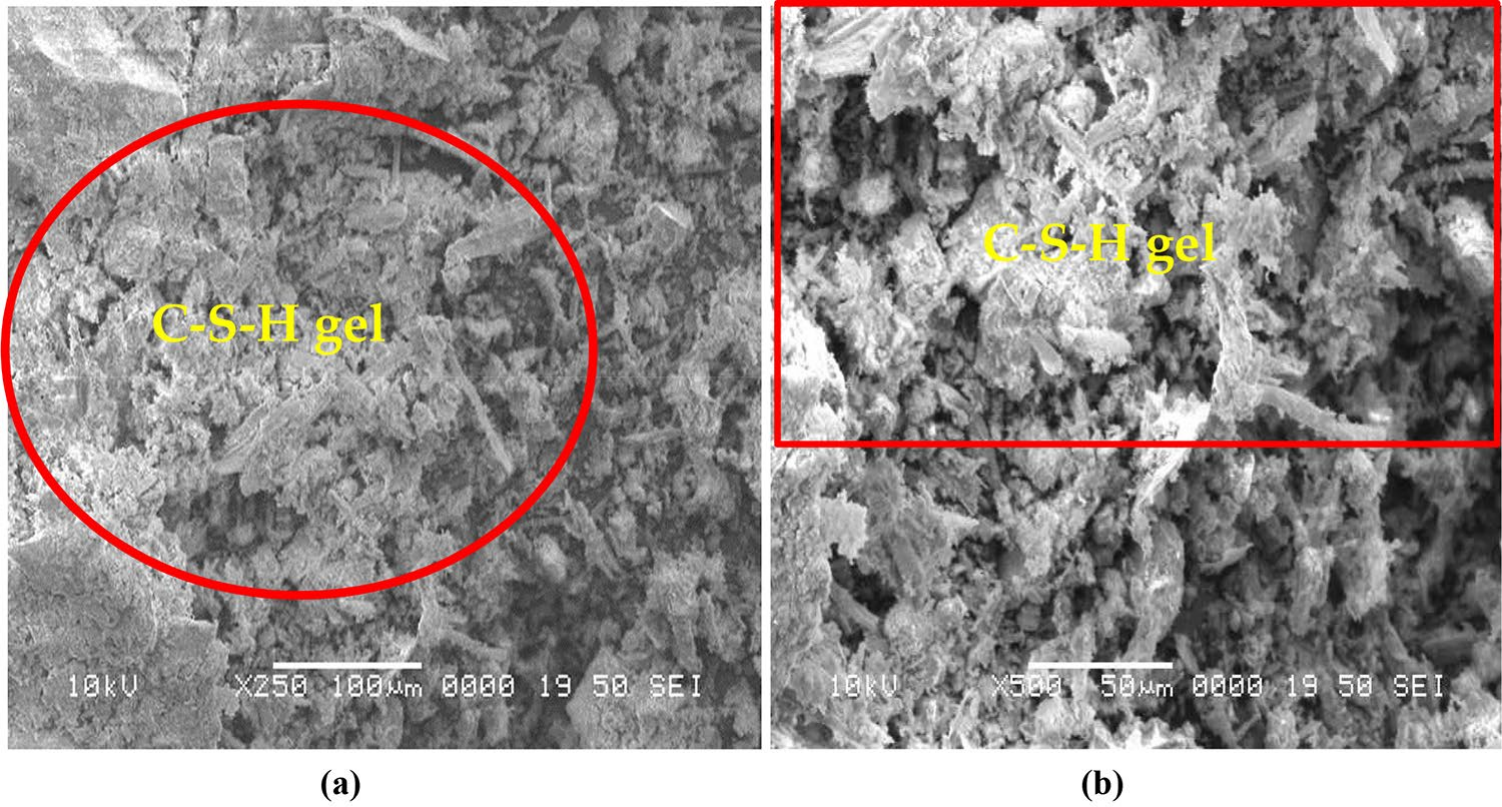
SEM of MHA at (**a**) 250X (**b**) 500X^71,76–78^.

Portland cement (PC) is locally available on the market, and it is used as a binding constituent for this study. However, the HH was collected from the barbershop and washed to remove dust. After removing the dust, HH used it in various lengths of 8–100 mm and diameters of 0.04–0.12 mm. The HH was used as fiber-reinforced material with various ratios of 0.5%, 1%, 1.5%, and 2% by volume fraction of concrete. The millet husk was collected and then burnt at a controlled temperature of 500–850 °C for six hours to produce ash. The residual ash was then left for 24 h at room temperature to cool down, and then the ash was sieved through a #4 sieve to eliminate unwanted ingredients. This sieved ash, MHA, was utilized in various replacement levels of 10%, 20%, 30%, and 40% as sand substitute constituents in concrete. The chemical composition of PC and MHA is shown in Table 1. X-ray powder diffraction (XRD) and scanning electron microscopy (SEM) are shown in Figs. 1 and 2, respectively. The microscopic characteristics of MHA, as seen through SEM, are shown in Fig. 2a,b. Under low magnifications (Fig. 2a), researchers look at C–H–S gel, which becomes more apparent when noticed at higher magnifications (Fig. 2b). Based on these pictures, it can be concluded that MHA includes particles that have the potential to act as pore fillers in concrete. Furthermore, the specimens underwent an X-ray examination to classify the distinct alterations in amorphous and crystalline silica at various temperatures. MHA burning may assess the specimen's crystal-like configuration by contrasting the small mirror image's intensity with a broad peak at 26 degrees. The specimen undergoes a contraction or expansion based on its processing circumstances, and the first stage is established by moving the peak either to the right or left^71^. We used natural hill sand as the fine aggregate (FA) with a size of 4.75 mm, and crushed stone as the coarse aggregate (CA) with a size of 20 mm. The sieve analysis of aggregates was conducted following ASTM C136^72^. FA and CA's specific gravity and water absorption were computed under ASTM C128-93^73^ and ASTM C127-93^74^, respectively. The bulk density of FA and CA was obtained following ASTM C29-97^75^. The assets of aggregates are tabulated in Table 2. The tap water was served in mixing and curing for this work.

**Table 1:** Chemical composition of PC and MHA.

Binder	Compound (%)						Specific Gravity
	SiO_2_	Al_2_O_3_	Fe_2_O_3_	CaO	Na_2_O	SO_3_	
MHA	69.40	5.80	3.36	10.50	1.10	1.85	2.25
PC	20.78	5.11	3.17	60.22	0.18	2.86	3.13

**Table 2:** Physical properties of aggregates.

Aggregates	Physical properties of aggregates			
	Specific gravity	Water absorption (%)	Bulk density (compacted)	Fineness modulus
Fine aggregates	2.62	1.25	1744 kg/m^3^	2.21
Coarse aggregates	2.66	0.55	1655 kg/m^3^	6.75

### Mix proportions

This experimental study investigated the fresh and mechanical properties of concrete that incorporated 0–40% MHA (with an increment of 10% and by weight of sand) as sand substituting material and was reinforced with 0–2% HH fibers (with an increment of 0.5% and by volume fraction of concrete) individually and in combination by following the ASTM C192/C192M-19^79^. Therefore, 25 mix proportions were designed with a mix ratio of 1:1.5:3 and a constant water-cement (w/c) ratio of 0.52. Out of 25 mix proportions, one reference or control mix was prepared without including MHA or HH fibers. Four mixes were prepared with 0.5%, 1%, 1.5%, and 2% HH fibers, whilst an additional four mixes were prepared with 10%, 20%, 30%, and 40% MHA. The remaining 16 mixes were prepared with MHA and HH fibers in combination. The various mix proportions are illustrated in Table 3.

**Table 3:** Mixture percentage of HHF concrete.

Mixture ID	PC (%)	Sand replacement (%)		Quantity of constituents used in concrete mixture (kg/m^3^)					
		FA	MHA	PC	MHA	FA	CA	HH Fiber (%)	Water
C	100	100	0	385	0	578	1155	0	200
HHF0.5	100	100	0	385	0	578	1155	0.5	200
HHF1	100	100	0	385	0	578	1155	1	200
HH1.5	100	100	0	385	0	578	1155	1.5	200
HHF2	100	100	0	385	0	578	1155	2	200
MHA10	100	90	10	385	57.8	520.2	1155	0	200
MHA20	100	80	20	385	155.6	462.4	1155	0	200
MHA30	100	70	30	385	173.4	404.6	1155	0	200
MHA40	100	60	40	385	231.2	346.8	1155	0	200
MHA10HHF0.5	100	90	10	385	57.8	520.2	1155	0.5	200
MHA20HHF0.5	100	80	20	385	155.6	462.4	1155	0.5	200
MHA30HHF0.5	100	70	30	385	173.4	404.6	1155	0.5	200
MHA40HHF0.5	100	60	40	385	231.2	346.8	1155	0.5	200
MHA10HHF1	100	90	10	385	57.8	520.2	1155	1	200
MHA20HHF1	100	80	20	385	155.6	462.4	1155	1	200
MHA30HHF1	100	70	30	385	173.4	404.6	1155	1	200
MHA40HHF1	100	60	40	385	231.2	346.8	1155	1	200
MHA10HHF1.5	100	90	10	385	57.8	520.2	1155	1.5	200
MHA20HHF1.5	100	80	20	385	155.6	462.4	1155	1.5	200
MHA30HHF1.5	100	70	30	385	173.4	404.6	1155	1.5	200
MHA40HHF1.5	100	60	40	385	231.2	346.8	1155	1.5	200
MHA10HHF2	100	90	10	385	57.8	520.2	1155	2	200
MHA20HHF2	100	80	20	385	155.6	462.4	1155	2	200
MHA30HHF2	100	70	30	385	173.4	404.6	1155	2	200
MHA40HHF2	100	60	40	385	231.2	346.8	1155	2	200

### Testing procedures

#### Slump test

It was performed on the 25 mixes of concrete reinforced with HH fibers by volume fraction, several percentages of MHA as a sand substitute, and combined use of HH fibers and MHA in concrete by following the ASTM C143-90^80^.

#### Hardened properties

**Figure 3:**
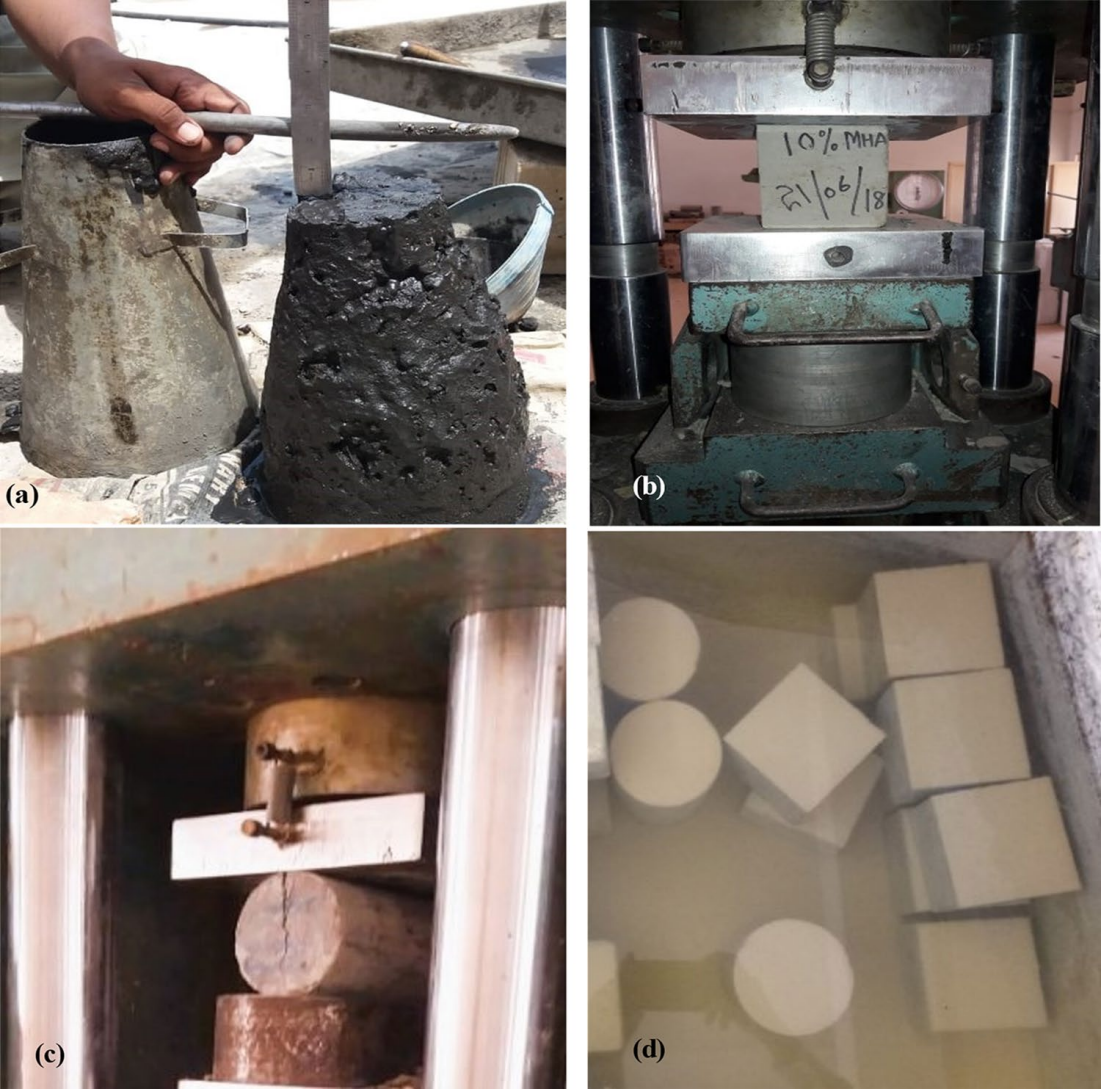
Investigational arrangement (**a**) Slump test, (**b**) Compressive strength test, (**c**) Splitting tensile strength, (**d**) Water curing tank.

The hardened properties of concrete were investigated to determine compressive strength, splitting tensile strength, and dry density. However, 75 cubical samples (100 mm × 100 mm × 100 mm) were prepared to determine the compressive strength of concrete incorporating several proportions of HH fibers and MHA after the specimens were cured for 28 days by obeying the ASTM C39/C39M^81^. The indirect tensile strength was determined on 75 cylinders (200 mm × 100 mm) after 28 days of curing following the guidelines of ASTM C 496-90^82^. Moreover, the density was performed on 75 cubical samples of concrete reinforced with various ratios of HH fibers, MHA as sand substitute ingredients, and combined use of HH fibers and MHA in concrete after 28 days of curing were obtained by following ASTM C1754/C1754M^83^. Furthermore, three concrete samples were used at every percentage of HH fibers and MHA separate and together at 28 days. Figure 3 depicts the testing arrangement.

## Results and discussions

### Slump test

**Figure 4:**
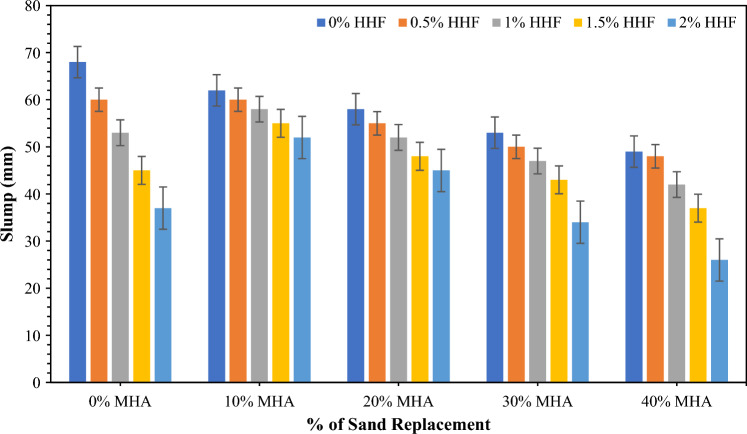
Slump test of eco-friendly concrete containing MHA and HH fibers.

Figure 4 presents the slump of concrete reinforced with 0.5–2% HH fibers and 10–40% of MHA as sand substitute ingredients separated and combined in concrete. However, the workability of concrete is measured by combining several proportions of HH. Reductions of 11.76%, 22.10%, 33.82%, and 45.60% were observed for the concrete mixes that incorporated 0.5%, 1%, 1.5%, and 2% of HH fibers, respectively, compared with that for control concrete without HH fibers. Thus, the flow of fresh concrete was restricted by an accumulation of HH fibers. This drop-in workability of concrete can be associated with the hygroscopic nature of HH which absorbs a greater quantity of water as the content of HH rises in concrete. This judgement is linked to Bheel et al.^30,84^, and a comparable observation was perceived in Meghwar et al.^85,86^. Besides, the slump test results for concrete mixes incorporating MHA as sand substituting material are shown in Fig. 4. The control sample achieved a slump value of 68 mm, and the workability was reduced with MHA incorporation. The MHA10 concrete mix achieved a slump value of 60 mm, which was 7.70% less than the control concrete mix. Further increase in MHA concentration reduced the workability even further. As a result, the lowest slump of 49 mm was achieved by concrete mix incorporating 40% MHA as FA replacement. This decrease in workability can be accredited to the MHA with a high specific surface area (High permeable) compared with other concrete components; thus, it requires more water^71^. Dayo et al.^87^ detected that the slump concrete dropped with the rise in sugarcane bagasse ash content used as FA in concrete.

Furthermore, Fig. 4 presents the slump of concrete reinforced with 0.5–2% of HH fibers by volume fraction of concrete along with 10–40% of MHA as a sand substitute ingredient in concrete. As observed, the combined utilization of HH fibers and MHA decreased workability, but the slump value varied with the variation in the combination. The lowest workability value of 26 mm was achieved for MHA40HHF2, which consisted of 40% MHA and was reinforced with 2% HH fibers. The further loss in workability of concrete incorporating MHA and HH fibers is expected owing to the specific surface area of MHA and HH fibers and the water absorption of MHA. A similar finding was perceived by Keerio et al.^88^ when they synergically used glass powder and silica fume as sand and cement substituting material, respectively. A comparable pattern was seen in the study conducted by Bheel et al.^89^.

### Dry density (DD)

**Figure 5:**
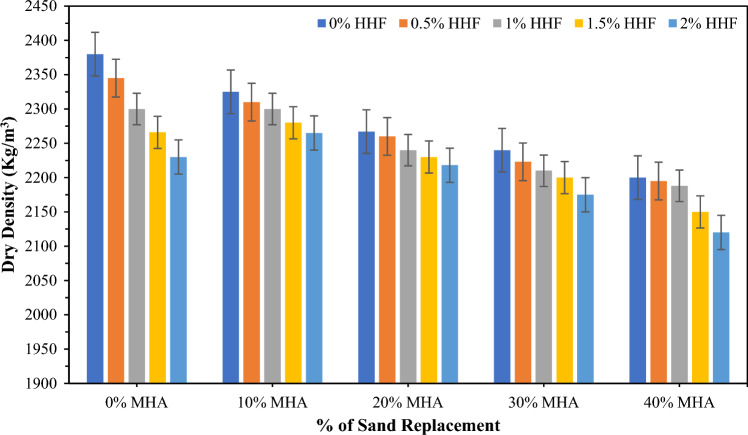
Dry density of concrete containing HH fibers and MHA.

Figure 5 displays the average dry density of concrete mixes that incorporated various HH fibers and MHA contents individually and together. However, the average dry density of the control concrete mix was determined to be 2380 kg/m^3^ after being subjected to water curing for 28 days. The dry densities were determined to be 2345, 2300, 226, and 2230 kg/m^3^ when 0.5%, 1%, 1.5%, and 2% HH fibers were used to reinforce the concrete. These values were 1.471%, 3.361%, 4.790%, and 6.3% less than the control concrete mix. The dry density declined with the growth in the volume of HH fibers, as displayed in Fig. 5. The drop in the dry density may be accredited to the lesser density of the HH fibers than those of other ingredients of concrete, and HH fibers used in concrete entrap extra air compared with control concrete without HH fibers. The decrease in dry density with HH fibers is similar to the reports of Bheel et al.^30^ and Meghwar et al.^86^. Besides, Fig. 5 indicates the dry density of concrete mixes that incorporated 10–40% MHA as sand substitute components in a concrete mixture at 28 days. As observed, the growth in MHA content declined the dry density of concrete. Dry densities of 2325, 2267, 2267, and 2200 kg/m^3^ were determined for concretes incorporating 10%, 20%, 30%, and 40% MHA, respectively. Thus, the loss in dry density ranged from 2.31 to 7.56%, including MHA content as FA replacement. This decrease in the dry density can be ascribed to the specific gravity of MHA (2.25), which is significantly lower than those of other ingredients of concrete (Cement = 3.13, FA = 2.62, and CA = 2.66). Similar findings were reported by Bheel et al.^71^. In addition, Fig. 5 displays the average dry density of concrete mixes that incorporated combined HH fibers and MHA contents. The dry density varied for different combinations of HH Fibers and MHA.

The highest dry density for the combination was 2310 kg/m^3^, and it was attained by MHA10HHF0.5 concrete mixture that combined 10% MHA and was reinforced with 0.5% HH fibers. In the meantime, the lowest dry density was noted by 2120 kg/m^3^, at MHA40HHF2 concrete mix that incorporated 40% MHA and reinforced with 2% HH fibers. Therefore, the collective utilization of MHA and HH fibers in concrete caused the loss in dry density ranging from 2.94 to 10.92% associated with the control mixture of concrete. This fall in the dry density can be ascribed to the lesser specific gravity of MHA and density of HH fibers than those of other concrete components. This opinion is accompanied by that of Bheel et al.^90^, who reported that the density was dropped as metakaolin (MK) and GGBFS as a binary cementitious component (BCM) improved in the concrete mixture. A similar trend was found in Bheel et al.^91^. Furthermore, this decline in the density of concrete combined with several concentrations of HH fibers and MHA as replacement of FA indicates that HH fibers and MHA are viable materials when a constraint to decline a dead load of concrete structures exists.

### Compressive strength (CS)

**Figure 6:**
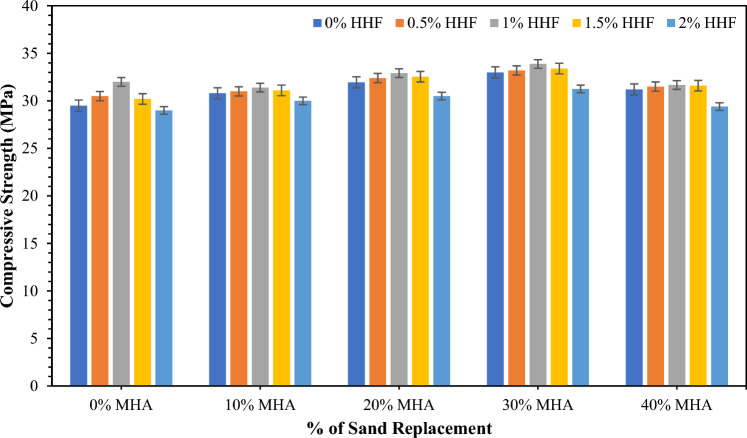
CS of concrete combined MHA and HH fibers.

Figure 6 illustrates the CS of hardened concrete mix reinforced with 0–2% of HH fibers and 10–40% of MHA at 28 days. The average CS of the control concrete mix was determined to be 29.5 MPa. The CS is augmented with the reinforcement of HH fibers. Specifically, 30.5 and 32 MPa were achieved by HHF0.5 and HHF1 concrete mixes that incorporated 0.5% and 1% HH fibers, respectively. However, a further increase in HH fiber volume decreased the CS of concrete. With the accumulation of 1.5% HH fibers, the concrete achieved a CS of 30.2 MPa, which was 2.38% higher than that of the control concrete (CM) samples. The CS dropped to 1.70% compared with that of the control concrete sample of 29 MPa with the addition of 2% HH fibers. As expected, the CS would improve with the accumulation of HH fibers because the fibers restrict the development of cracks owing to the fibers–cement mixture interfacial bonding and the fracture-bridging action of fibers. However, the drop in CS may be attributed to the agglomeration of fiber. This observation is associated with Bheel et al.^30^, who studied that the strength was boosted up to 1% of HH fibers and then reduced with the increase in HH fibers over 28 days. However, Fig. 6 reveals the compressive strength of concrete, including 0–40% MHA as a sand substitute component at 28 days. As observed, the addition of MHA as a sand substitute material was beneficial in gaining strength. The concrete achieved average CS of 30.8, 31.95, 33, and 31.20 MPa when 10%, 20%, 30%, and 40% MHA were used as sand substitutes. Compared with the CM sample, they accounted for 4.41%, 8.31%, 11.86%, and 5.76% increases in CS. The enhancement in CS of concrete when sand is substituted with MHA can be ascribed to the pozzolanic reaction. MHA is a pozzolanic material according to its chemical composition. Thus, it triggers the pozzolanic reaction, during which the accessible Ca(OH)_2_ is used, and a secondary C–S–H solution is formed. This C–S–H solution is accountable for gaining strength. However, the decrease in CS when 40% MHA is utilized can also be associated with the pozzolanic reaction. Concrete only produces approximately 22% calcium hydroxide, which is freely available^92^. Thus, this calcium hydroxide is consumed to produce maximum C–S–H for 30% MHA. As for concrete incorporating 40%, the calcium hydroxide is insufficient. Therefore, the pozzolanic reaction is restricted. Thus, limited C–S–H gels are formed, which ultimately reduces the strength. This judgment is associated with Bheel et al.^71^, who showed that the CS was augmented with MHA growth as a cementitious component to 10% after 90 days. Keerio et al.^88^ documented that the strength was boosted with the growth in glass powder as a sand substitute to 30% on 28 days. A similar trend was observed in Kanaka and Thiyagarajan^93^ and Bajad et al.^94^.

Figure 6 demonstrates the average CS of concrete incorporating HH fibers and MHA in different combinations. The highest CS for the combinations was 33.88 MPa, which was achieved by MHA30HHF1 concrete that incorporated 30% MHA and was reinforced with 1% HH fibers. In the meantime, the lowest CS amongst the different combinations was 29.40 MPa, which was achieved by MHA40HHF2 concrete that incorporated 40% MHA and was reinforced with 2% HH fibers. The difference in CS ranged from − 0.34% to 14.85%. The gain in strength can be accredited to the bridging mechanism of the HH fibers and the development of secondary C–S–H gels through the pozzolanic reaction triggered by the MHA. This observation is interrelated to Bheel et al.^90^, who indicated that the strength was boosted with GGBFS and MK as BCM in concrete to 10%. The maximum CS was achieved by 33.88 MPa at MHA30HHF1 in concrete at 28 days. However, the maximum recycling of waste materials is beneficial because of natural resource depletion. Therefore, MHA40HHF2, which attained slightly lesser compressive strength than the CM sample, can be taken as the optimum mixture of concrete.

### Splitting tensile strength (STS)

**Figure 7:**
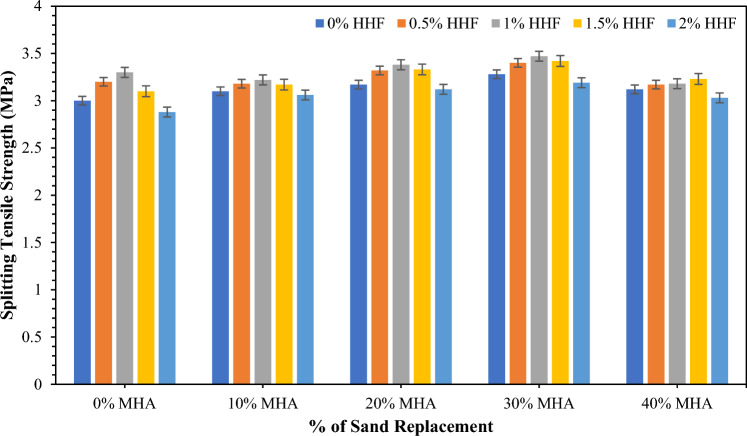
STS of concrete combined with MHA and HH fibers.

Figure 7 presents the average indirect tensile strength of concrete reinforced with different dosages of HH fibers and 10–40% of MHA at 28 days. The control concrete exhibited an indirect tensile strength of 3 MPa. The addition of HH fibers increased the splitting tensile strength. In particular, 3.2, 3.3, 3.1, and 2.88 MPa were achieved by concrete samples reinforced with 0.5%, 1%, 1.5%, and 2% HH fibers. The increase in splitting tensile can be ascribed to the bridging mechanism of the fibers, which restricts the tensile strength whilst simultaneously preventing the propagation of cracks. Bheel et al.^30^ observed that the STS augmented when HH fibers were used up to 2%. However, the current study found that the strength of concrete is obtained maximum by utilizing 1.5% HH fiber reinforcement. Then the strength reduces due to the agglomeration of fiber. However, Fig. 7 reveals the STS of concrete, including different MHA contents. The replacement of sand with MHA content in concrete is augmented by the STS. Specifically, 3.1, 3.17, 3.28, and 3.12 MPa were achieved by concrete samples incorporating 10%, 20%, 30% and 40% MHA, correspondingly. As explained in the earlier section, the gain in strength can be associated with developing secondary C–S–H gels through the pozzolanic reaction. Bheel et al.^71^ also used MHA as a cementitious substituting material. They found that 15% of MHA successfully achieved higher STS than that of CM. Keerio et al.^88^ observed that the STS was enhanced as glass powder was utilized as sand substituting material and found that up to 30% glass powder could be used.

The average indirect tensile strength of concrete incorporating HH fibers and MHA in different combinations is revealed in Fig. 7. The lowest STS amongst the different combinations was 3.03 MPa, achieved by MHA40HHF2 concrete that incorporated 40% MHA and was reinforced with 2% HH fibers. In the meantime, the maximum STS amongst the different combinations was 3.47 MPa, which was achieved by MHA30HHF1 concrete that incorporated 30% MHA and was reinforced with 1% HH fibers. The gain in STS ranged from 1 to 15.67%. The increase in STS can be accredited to the bridging mechanism of the HH fibers and the development of secondary C–S–H gels through the pozzolanic reaction triggered by the MHA. This observation is associated with Bheel et al.^90^, who found that the STS was boosted as the amount of GGBFS and MK as BCM in concrete was equal to 10%. The maximum STS is attained by 3.47 MPa at MHA30HHF1 concrete. However, the maximum recycling of waste materials is beneficial because of the depletion of natural resources. Therefore, MHA40HHF2, which achieved lower STS than the MHA30HHF1 concrete, is higher STS than the CM sample; therefore, it can be measured as the optimum concrete mix in terms of STS.

### Cost analysis of MHA

In order to assess the feasibility of using MHA as a substitute for sand in concrete, it is necessary to calculate and analyse its impact on the overall cost of the concrete. Despite the MHA being a waste material collected at no cost, there are still expenses associated with its processing. The price includes the transportation expenses incurred in transporting the raw material from its origin to the facility for blending concrete. The MHA was subjected to the processes of drying, grinding, and screening, all of which resulted in additional costs. The current electricity usage rate is $0.046 per kilowatt-hour, while the cost of one litre of diesel is $0.44. Table 4 displays the comprehensive expense of handling the MHA, while Table 5 demonstrates the acquisition of the sand material from a nearby supplier.

**Table 4:** Cost used for MHA processing.

Material	Transportation of 1000 kg material			Electricity used for 1000 kg material (kWh)			
	Distance (km)	Consumption of diesel (L/10 km)	Cost of diesel ($/L)	Electricity consumed per kWh		Cost of electricity ($/kWh)	Total cost ($/kg)
				Drying	Grinding and sieving		
MHA	15	1	0.44	18	71.80	0.046	0.0048

**Table 5:** Cost of ingredients used in the research.

Materials	MHA	Sand
Cost ($/kg)	0.0048	0.009

## RSM modelling

The use of Response Surface Methodology (RSM) as an optimization tool for examining practical properties has notable advantages over other methodologies, particularly in contrast to techniques such as the Taguchi method. The efficiency of RSM is derived from its capacity to provide substantial outcomes with a reduced number of experimental iterations, therefore preserving resources and accelerating the research procedure. Furthermore, RSM's flexibility in modeling allows for the detailed depiction of nonlinear connections between input factors and output responses, which is critical for gaining a thorough understanding of tangible characteristics. Furthermore, the emphasis on local optimization in RSM enables accurate fine-tuning towards optimum solutions, which is a vital benefit in real research where even small alterations can have a significant impact on outcomes. The enhanced utility of RSM models is attributed to their interpretability, which enables researchers to effectively identify and analyze the individual and interaction impacts of components on certain qualities. By combining Response Surface Methodology (RSM) with carefully constructed experimental frameworks, a methodical investigation of the design space guarantees reliable and applicable results. To summarize, RSM is a reliable and effective method for improving the properties of rubberized concrete. It provides a complete approach that combines the flexibility of modeling, the efficiency of experimentation, and the capacity to understand results.

### Analysis of variance (ANOVA)

Response surface modeling (RSM) is the method employed to build the algorithms, and analysis of variance (ANOVA) is applied to assess them. Nevertheless, the mechanical properties of concrete mixes containing 0–40% MHA as sand replacement and various amounts of HHF were studied for RSM modeling and optimization. Additionally, it was determined that linear models were better suited for slump tests and dry density responses, but quadratic approaches were preferred for compressive strength and splitting tensile strength. Additionally, Eqs. (1) through (4) encode each of these replies.

It is possible to predict the outcome of changing parameter values by using equations specified in terms of coded constituents. The standard representation of the constituent levels is + 1 for the greatest levels and − 1 for the least favorable levels. The coded equations could be applied to get a measure of the corresponding significance of the parameters by applying the factor coefficients. MHA and HHF are input parameters, along with A and B. Table 6 contains the ANOVA's findings.

**Table 6:** Outcomes of ANOVA.

Response	Source	Sum of squares	Df	Mean square	F-Value	*p* value > F	Significance
Slump Test	Model	965.43	2	482.71	16.98	0.0002	Yes
	A-MHA	515.83	1	515.83	18.15	0.0009	Yes
	B-HHF	602.39	1	602.39	21.19	0.0005	Yes
	Residual	369.51	13	28.42			
	Lack of fit	369.51	8	46.19			
	Pure error	0.50	5	0.100			
Dry Density	Model	49028.88	2	24514.44	66.78	< 0.0001	Yes
	A-MHA	41676.33	1	41676.33	113.53	< 0.0001	Yes
	B-HHF	13712.86	1	13712.86	37.36	< 0.0001	Yes
	Residual	4772.06	13	367.08			
	Lack of fit	4772.06	8	596.51			
	Pure error	242.00	5	48.40			
	Cor total	53800.94	15				
CS	Model	23.13	5	4.63	17.20	0.0001	Yes
	A-MHA	1.44	1	1.44	5.34	0.0434	Yes
	B-HHF	2.05	1	2.05	7.61	0.0202	Yes
	AB	0.78	1	0.78	2.89	0.1200	No
	A^2^	3.84	1	3.84	14.30	0.0036	Yes
	B^2^	9.63	1	9.63	35.81	0.0001	Yes
	Residual	2.69	10	0.27			
	Lack of fit	2.69	5	0.54			
	Pure error	2.05	5	0.50			
	Cor total	25.82	15				
STS	Model	0.30	5	0.060	20.24	< 0.0001	Yes
	A-MHA	0.022	1	0.022	7.48	0.0210	Yes
	B-HHF	0.024	1	0.024	8.12	0.0173	Yes
	AB	9.307E-006	1	9.307E-006	3.154E-003	0.9563	No
	A^2^	0.095	1	0.095	32.12	0.0002	Yes
	B^2^	0.089	1	0.089	30.03	0.0003	Yes
	Residual	0.030	10	2.951E-003			
	Lack of fit	0.030	5	5.902E-003			
	Pure error	0.015	5	0.0038			
	Cor total	0.33	15				

The ANOVA significance level was set at 95%, and a prototype component with a possibility of a smaller amount than 5% is deemed important. By the way, all generated prototypes are statistically essential since their probabilities are smaller than 0.05. The statistically significant value of the mathematical model elements A, B, AB, A^2^, and B^2^ pertains to the CS model. The key model parameters for STS are thus A, B, AB, A^2^, and B^2^. For the slump test and the dry density model, A and B are also important model variables.

The determination coefficient (R^2^) serves as a substantial measurement of efficiency and effectiveness. The R^2^ statistic represented as a percentage ranging from 0 to 1, assesses the degree of precision between the model and the actual information. Fit is proportional to value; conversely, a superior fit corresponds to a lower value. Table 7 contains R^2^ and additional assessments of model factors. R^2^ values for slump test, dry density, CS, and STS were 72.32 percent, 91.13 percent, 89.58 percent, and 91 percent, correspondingly. Moreover, "Adeq. Precision" calculates the signal-to-noise ratio. A ratio bigger than 4 is desired. According to Table 7, the Adeq. The precision values for the slump test, dry density, compressive strength, and tensile strength models are 14.53, 27.27, 11.20, and 14.10 respectively. These results suggest that the mathematical frameworks are capable and could be utilized for predicting responses with accuracy.

**Table 7:** Assessment of model components.

Model validation constraints	Slump	DD	CS	STS
Std. Dev	5.33	19.16	0.52	0.054
Mean	48.94	2235.94	31.29	3.18
C.V. %	10.89	0.86	1.66	1.71
PRESS	650.14	8709.01	6.16	0.074
-2 Log likelihood	95.64	136.57	16.87	-55.33
R-squared	0.7232	0.9113	0.8958	0.9101
Adj R-squared	0.6806	0.8977	0.8438	0.8651
Pred R-squared	0.5130	0.8381	0.7616	0.7736
Adeq precision	14.536	27.270	11.202	14.107

1 $$Slump=+49.95-7.84\times A-8.94\times B$$

2 $$DD=+2243.88-70.47\times A-42.65\times B$$

3 $$CS=+32.76+0.42\times A-0.56\times B-0.40\times AB-1.18\times {A}^{2}-1.80\times {B}^{2}$$

4 $$STS=+3.36+0.052\times A-0.060\times B-1.390E-003\times AB-0.19\times {A}^{2}-0.17\times {B}^{2}$$

**Figure 8:**
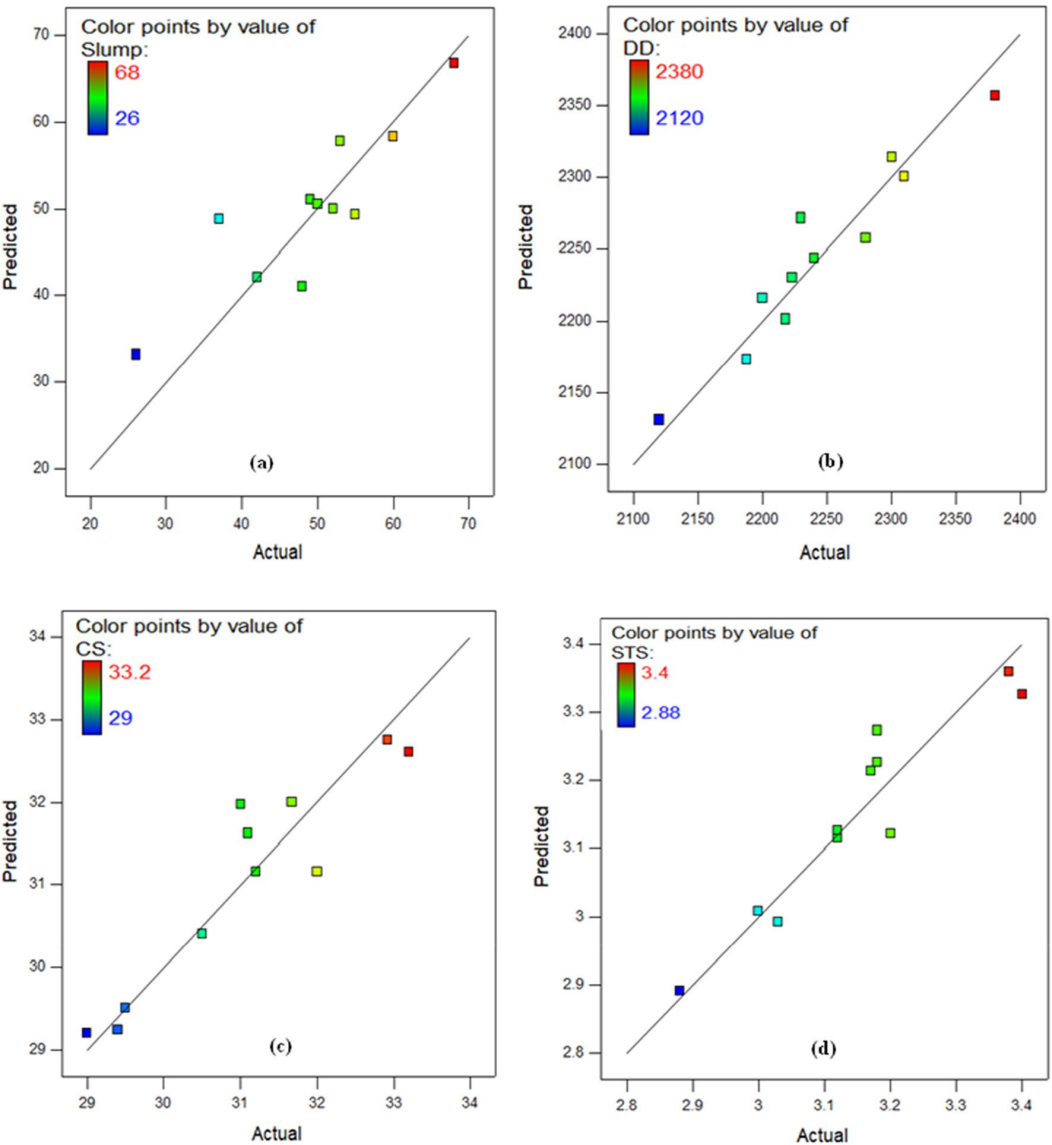
Predicted versus actual plot for (**a**) Slump test, (**b**) Dry density, (**c**) CS, and (**d**) STS.

The "Actual against Predicted" plot, as illustrated in Fig. 8 for the seven variables, is utilised as a powerful model diagnostic tool to more quickly analyse the condition and applicability of the created response models (slump, DD, CS, and STS). The linearity of the data sets along the fit line is shown in all charts to demonstrate the correctness of the models generated. The organization of data facts on plots implies that it is preferable that the error ingredients be regularly scattered^95–97^.

**Figure 9:**
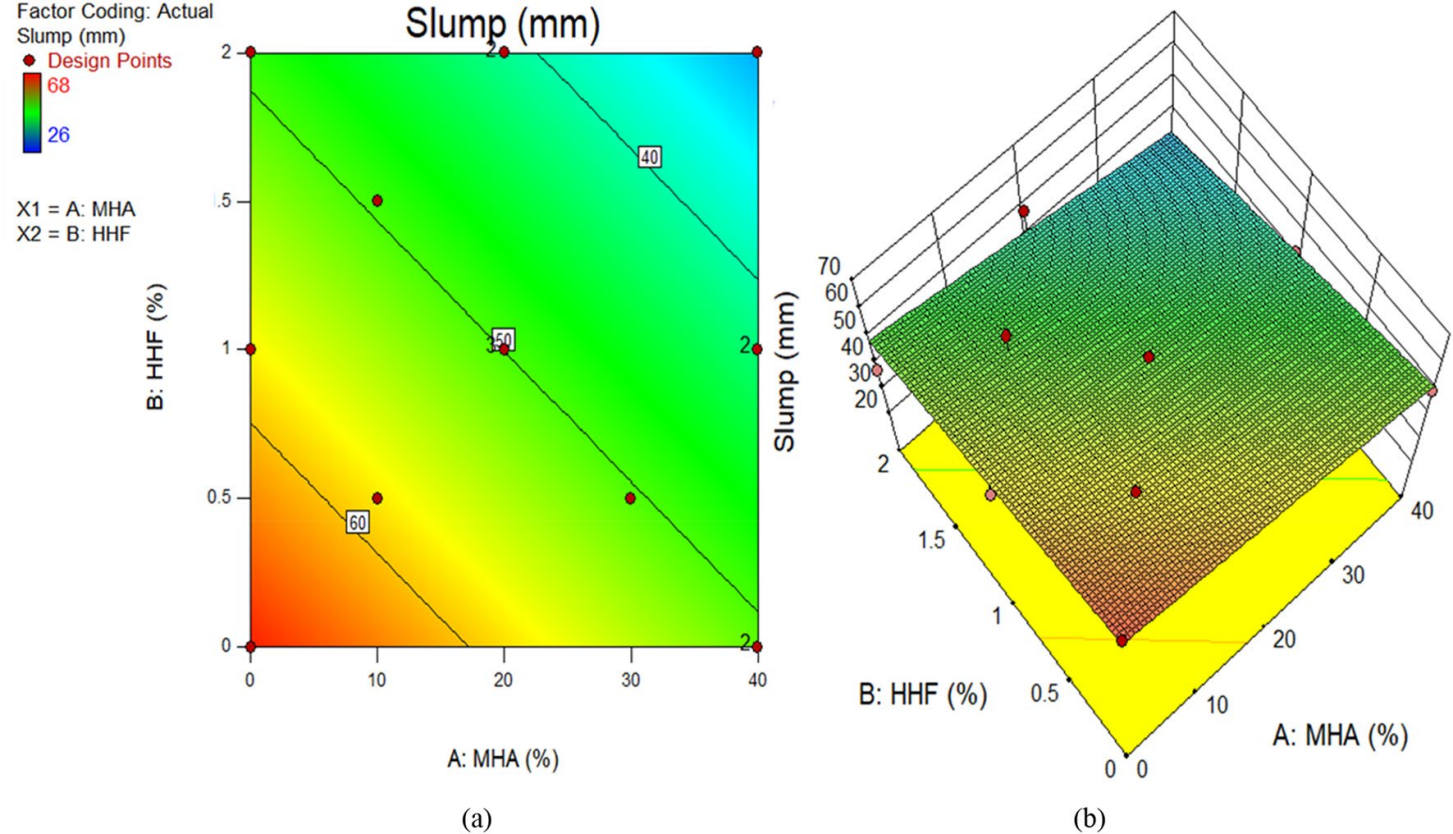
Slump test of concrete plots for (**a**) 2D and (**b**) 3D graphs.

**Figure 10:**
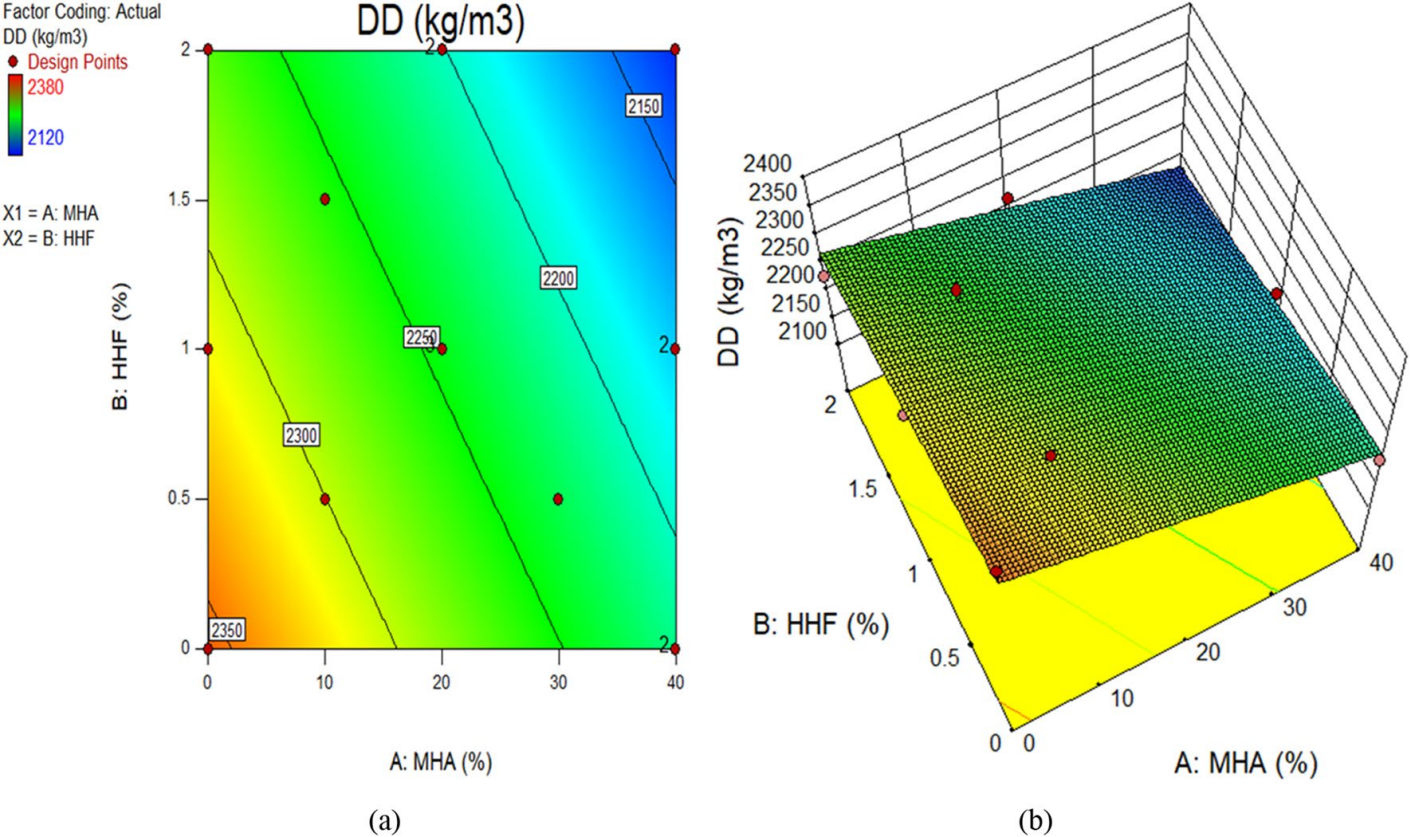
Dry density of concrete plots for (**a**) 2D and (**b**) 3D graphs.

**Figure 11:**
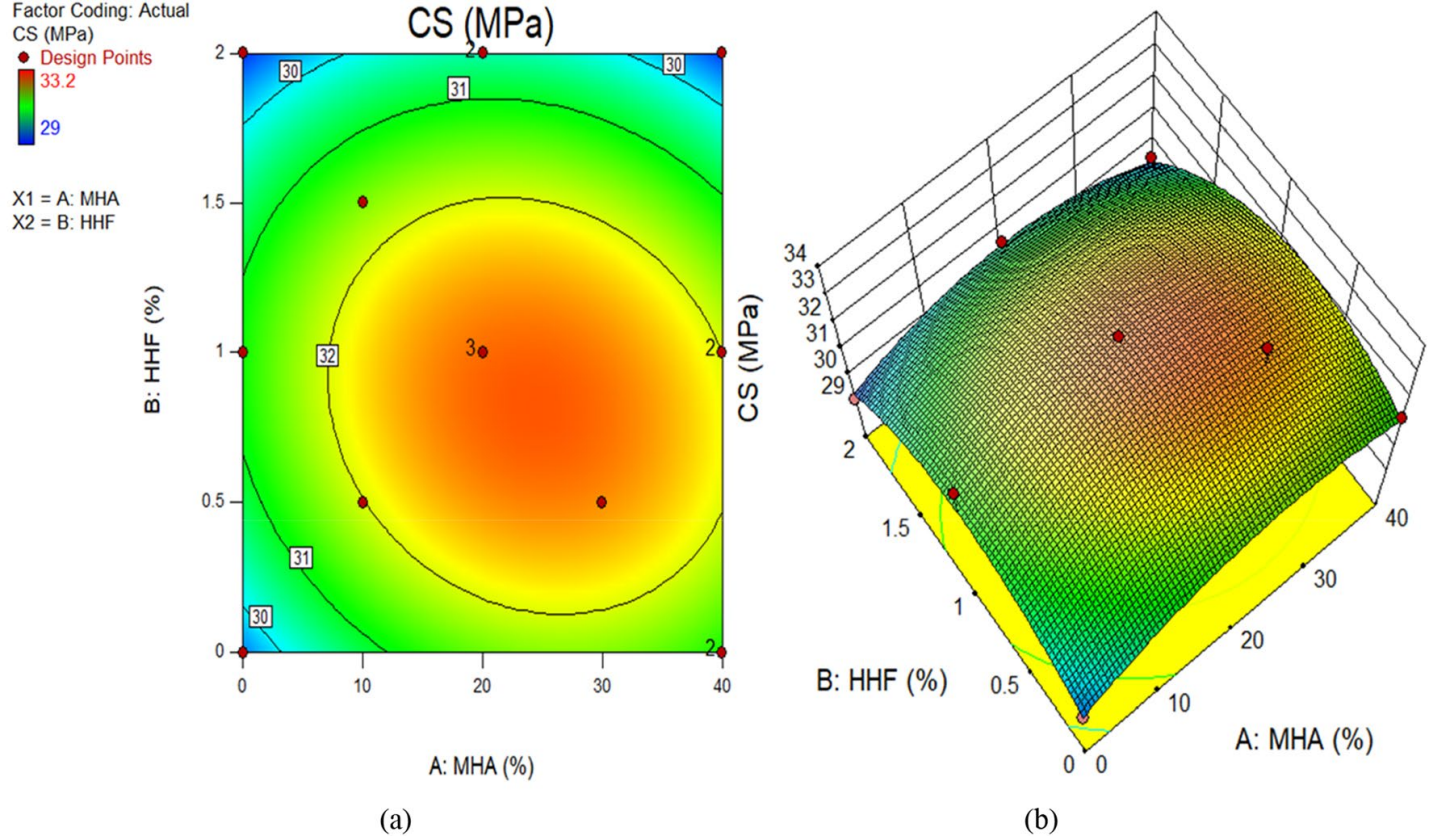
CS of concrete plots for (**a**) 2D, and (**b**) 3D graphs.

**Figure 12:**
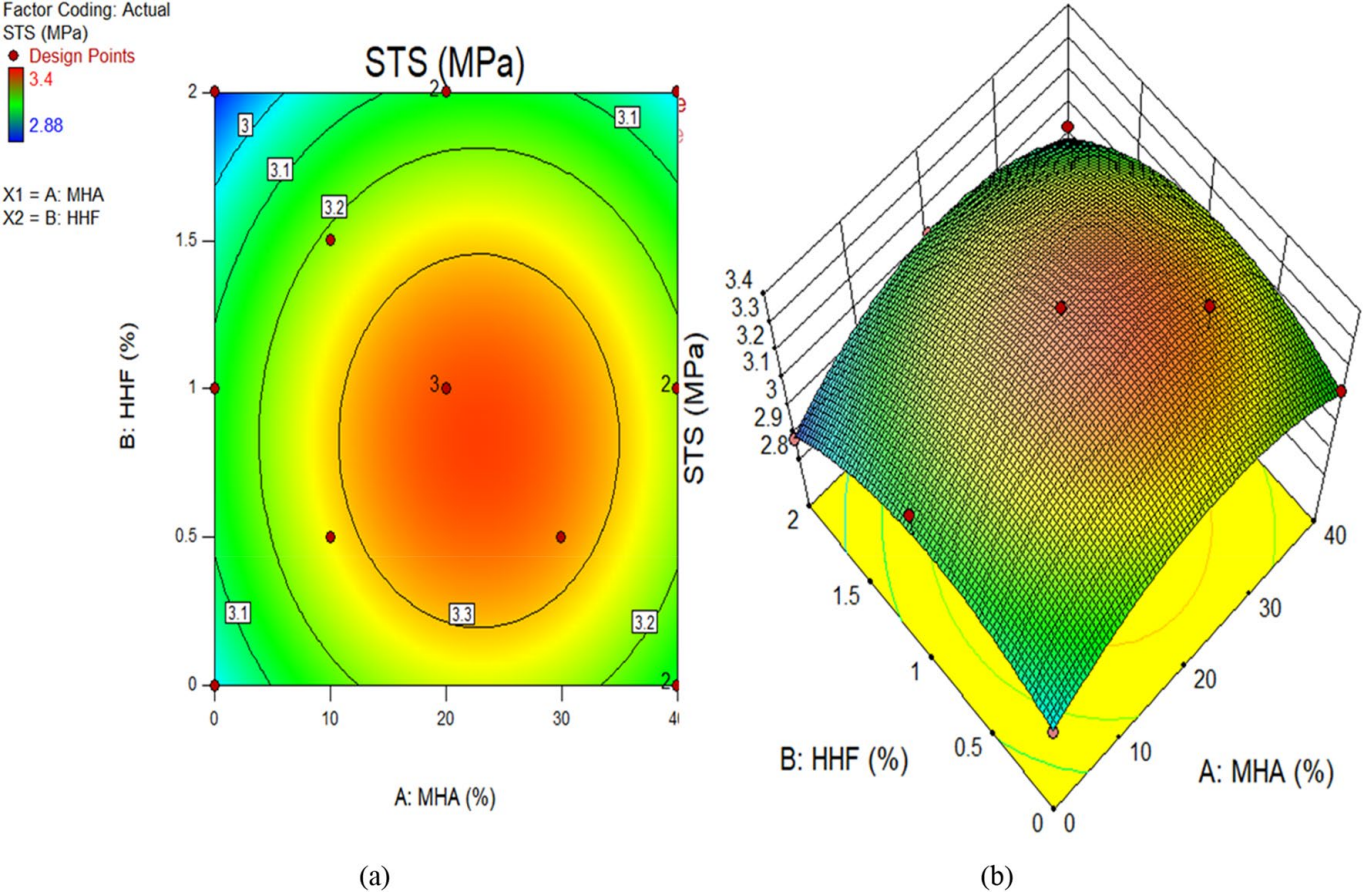
STS of concrete plots for (**a**) 2D, and (**b**) 3D graphs.

The impact of the interaction between the input parameters on the outputs is shown by using contour plots in two dimensions and response surface graphs in three dimensions. Figures 9, 10, 11, 12 show the response surface graphs for the Slump model, the DD model, the CS model, and the STS model, respectively. For example, when there are two input parameters, as there are in this investigation, the interaction between the variables may be illustrated. In this scenario, both two-dimensional contour plots and three-dimensional response graphs for the HHF and MHA interaction are shown.

The color coding of the graphs indicates the magnitude of the response and the different input factor values under consideration. Figure 9a and b demonstrate, respectively, the 2D contour and 3D response graphs for slump. The figures demonstrate that high intensity of slump was recorded in the control combination, as well as up to 1.22 percent HHF and 27.96 percent MHA as sand replacement material. It has been shown that the slump of concrete decreases with increasing amounts of HHF and MHA in the mixture. Similar responses were seen at the surface for dry density. Moreover, Fig. 11 and b exhibit, respectively, the 2D contour and 3D response graphs for CS. As a sand replacement material, a high concentration of CS has been reported up to 1.22 percent of HHF and 27.96 percent of MHA, according to the graphs. In addition, the strength value is greatly enhanced by including up to 27.96 percent MHA and 1.22 percent HHF in the concrete. This is a result of MHA's void-filling and densification properties as a combination of pozzolanic materials. The remaining response surface diagrams for splitting tensile strength behave in the same way.

### Optimization

An optimization approach is performed to achieve the ideal values of the independent components that will achieve the intended output at its highest level. To attain the stated purpose, this is done by opting for objectives for quality and applying a variety of criteria and measures of relevance. These results suggest that the mathematical frameworks are capable and could be utilised for predicting responses with accuracy. Utilizing the desirability value (0 ≤ *dj* ≤ 1), the optimization is assessed. Achieving a more favourable result is directly proportional to the value approaching unity^98,99^.

**Figure 13:**
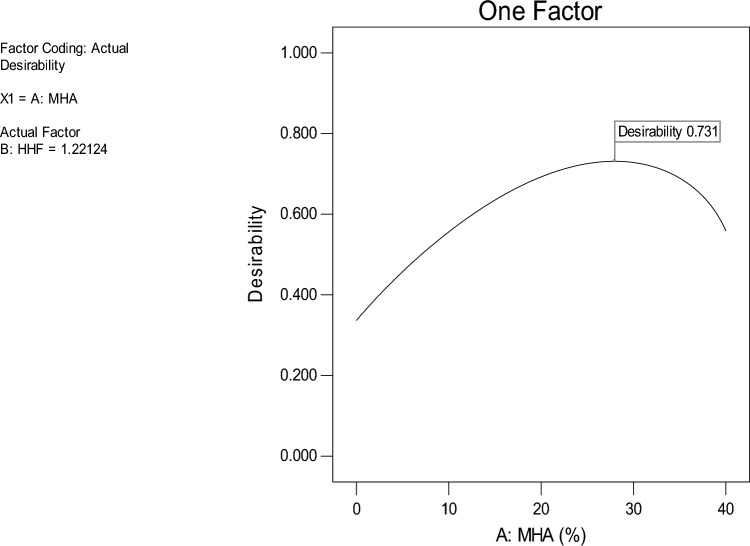
One factor response surface diagram for the desirability of the optimization.

Table 8 describes the goals and requirements for the optimization of this situation. According to Table 8, the goal of optimization is to maximize three output responses while decreasing the four output responses. However, the use of MHA has been limited to a range of 0–40%, and the usage of HHF has been limited to a range of 0–2%, so that the system can identify the ideal quantity necessary to attain the stated objective. The optimization results suggested that the maximum values for slump, DD, CS, and STS could be achieved by mixing 27.96 percent MHA and 1.22 percent HHF fibers in concrete. These values are 44.84 mm, 2206.32 kg/m3, 32.49 MPa, and 3.32 MPa correspondingly. The optimization's desirability is found to be 73.10 percent, which is appropriate assuming the nature of the significant response value variability. Figure 13 shows the one-factor response surface diagram for desirability.

**Table 8:** Optimization objectives and outcomes.

Factors		Input factors				Responses (Output Factors)	
		MHA (%)	HHF (%)	Slump (mm)	DD (kg/m^3^)	CS (MPa)	TS (MPa)
Value	Minimum	0	0	26	2120	29.0	2.88
	Maximum	40	2	68	2380	33.20	3.40

Goal		Range	Range	Minimize	Minimize	Maximize	Maximize
Optimization results		27.96	1.22	44.84	2206.32	32.49	3.32
Desirability						0.731 (73%)

In order to calculate the slump, DD, CS, and STS values after 28 days, experimental validation was completed by producing concrete specimens using optimal input parameters suggested by optimization. Table 9 displays the outcomes of the conducted examination. This demonstrates that the generated response approach is robust and may be applied to predict outcomes with a satisfactory degree of precision. As revealed in Table 9, the investigational error between practical and predicted findings was determined for each response using Eq. (5), and the outcomes are less than 6%, which is the permissible limit.

5 $$\delta =\left|\frac{{\vartheta }_{e}-{\vartheta }_{p}}{{\vartheta }_{p}}\right| \times 100$$

where $\delta$ represents the percentage error,* ϑ*_*e*_ indicates the experimental outcomes and* ϑ*_*p*_ displays the predicted values.

**Table 9:** Experimental validation.

Responses	Predicted value	Experimental value	Error (%)
Slump (mm)	44.84	47	4.82
DD (kg/m^3^)	2206.32	2238	1.44
CS (MPa)	32.49	33.12	1.94
STS (MPa)	3.32	3.36	1.20

## Conclusions

This work produced unique eco-friendly concretes using HH fibers as reinforcing material and MHA as a replacement for sand. Overall, combined use performed better than separate usage. The following are the conclusions drawn from the properties of fresh and mechanical concrete:

- The workability decreases with the increase in the HH fibers and MHA content separate and combined in concrete. This reduction is associated with the specific surface area of MHA and the water absorption of HH fibers.
- The density of concrete decreases with higher levels of HH fibers and MHA concentrations separate and combine in concrete. This drop in density is associated with the lower specific gravity and density of MHA and HH fibers than other concrete components.
- The compressive and tensile strengths were increased when the used of 30% of MHA and 1% of HH fibers together in concrete. The improvement in strengths can be attributed to the bridging mechanism of the HH fibers and the development of secondary C–S–H gels through the pozzolanic reaction triggered by the MHA.
- Response surface models were created using ANOVA to predict mechanical characteristics. The R2 value ranges from 72 to 91%. The use of 27.96% MHA and 1.22% human hair in concrete resulted in a 73% desirability factor.
- According to the research, 30% MHA and 1.5% HH fibers in concrete are the most effective solutions for structural purposes, taking into account resource depletion and waste material accumulation.

## Research limitations

The study on using HH fibers and MHA as replacements for sand in concrete discovered many problems in evaluating the characteristics of both fresh and hardened concrete. Some of the constraints encompass:

- Limited Generalization: The research may have concentrated on particular ratios and circumstances for integrating HHF and MHA, making it difficult to generalize the results to a wider variety of concrete compositions or environmental situations.
- Short-Term Evaluation: The study might be constrained by a comparatively short-term assessment of the concrete characteristics. The long-term consequences, including the durability and performance over an extended period, were not adequately examined.
- Environmental Variability: The study's environmental circumstances may not have included the whole range of real-world situations. Potential oversight may have occurred regarding the comprehensive evaluation of temperature, humidity, and other environmental variables.
- Limited Mechanical Property Assessment: The assessment of mechanical characteristics may have failed to include every relevant variable, such as fatigue resistance, impact strength, or other specialized performance requirements.

## Recommendations for future research

The study examining the utilization of HH fibers and MHA as replacements for sand in concrete suggests numerous suggestions for future research to improve the comprehension and utilization of these environmentally benign materials. Below are a few recommendations:

- Long-term durability Studies: Perform extensive, extended-term durability analyses to evaluate the effectiveness of concrete containing HHF and MHA over prolonged durations. Assess the resistance of materials to environmental variables, including freeze–thaw cycles, chemical exposure, and weathering.
- Optimization of Mix Proportions: Optimize the mix proportions of HH fibers and MHA by investigating a wider range of mix proportions. This will enable the identification of optimum combinations that provide superior characteristics in both fresh and hardened states. This may include using a methodical technique to determine the optimal ratios for various concrete applications.
- Life Cycle Assessment (LCA): Conduct a life cycle assessment (LCA) to examine the environmental impact of concrete mixtures that include HH fibers and MHA. Take into account the whole life cycle, such as the collection of raw materials, the manufacturing process, transportation, and disposal at the end of its life.
- Innovative Applications: Exploration of novel uses for HH fibers and MHA concrete that go beyond conventional structural components. Explore potential applications for integrating these materials into specialized construction projects or as components of sustainable building techniques.

By implementing these suggestions, future studies may enhance the comprehension of the possible advantages and difficulties linked to the use of HH fibers and MHA in concrete, hence promoting their broader acceptance in the construction sector.

## Novelty of the research

This research is unique because it investigates sustainable options for traditional concrete elements by integrating human hair fiber (HHF) and millet husk ash (MHA) as replacements for sand. The distinct amalgamation of these substances in different ratios not only adds to the environmental sustainability of the concrete but also introduces a novel method for improving the mechanical characteristics of concrete. The study delves into the synergistic impacts of HHF and MHA, beyond standard sand substitutes, to examine the possibility of these unconventional materials enhancing the overall performance of concrete. The study's focus on sustainable building techniques, together with a thorough examination of the unique concrete's mechanical and fresh qualities, establishes it as a groundbreaking endeavour in promoting environmentally friendly and efficient options in the construction sector.

## Data Availability

The datasets used and/or analyzed during the current study are available from the corresponding author upon reasonable request.
